# Piscida Erythrina—Jamaica Dogwood

**Published:** 1880-07

**Authors:** 


Piscida Erythrina—Jamaica Dogwood.—This new narcotic comes to us highly recommended, and is said to produce narcotic effects which are refreshing, and not followed like opium by hyperæmia of the brain, nausea and general nervous disturbance. It is said to be of value in bronchitis, asthma, nervous cough, writer's cramp, spasms of muscles due to functional causes, chorea, tetanus and especially in toothache to relieve pain. Dr. Isaac Ott, in the Detroit *Lancet*, June, 1880, gives the results of his studies concerning the physiological action of this drug, as follows:

1. It is narcotic to frogs, rabbits and men.
2. It does not effect the irritability of the motor nerves.
3. It does not attack the peripheral ends of the sensory nerves.
4. It produces a tetanoid state by a stimulant action on the spinal cord, and not by a paralysis of Setschenow's centers.
6. It dilates the pupil, which dilation passes into a state of contraction upon the supervention of asphyxia.
7. It is a salivator.
8. It increases the secretion of the skin.
9. It reduces the frequency of the pulse.

10. It increases arterial tension by stimulation of the monarchical vaso-motor center.

11. This increase of pressure is soon succeeded by a fall, due to a weakening of the heart itself.

If the action of piscidia is compared with that of chloral, it is found that the former has no dangerous action on the heart like the latter, nor such an energetic action like the latter upon the respiratory apparatus.

Compared with atropia, piscidia, unlike the former, does not paralyze the motor nerves; it does paralyze the cord or tympani; it does not arrest the sudoral secretion; it does not paralyze the pneumogastrics, and does not elevate greatly the arterial tension, but like it, it dilates the pupil. Compared with morphia, like it, it produces sleep, heightened excitability, spinal convulsions, general paralysis and stimulation of the main vaso-motor center; unlike it it dilates the pupil. In the use of this drug, I would like to add the caution that its surface is pleasure and its depth death.

